# Technological Developments and Strategic Management for Overcoming the COVID-19 Challenge within the Hospital Setting in Israel

**DOI:** 10.5041/RMMJ.10417

**Published:** 2020-07-31

**Authors:** Aharon (Ronnie) Abbo, Asaf Miller, Talya Gazit, Yonatan Savir, Oren Caspi

**Affiliations:** 1 Department of Cardiology, Rambam Health Care Campus, Haifa, Israel; 2 Medical Intensive Care Unit, Rambam Health Care Campus, Haifa, Israel; 3 Col. (res.) and Former Head of “MAMRAM,” The Technology and Cyber Defense Unit, Israel Defense Forces, Ramat Gan, Israel; 4 The Ruth & Bruce Rappaport Faculty of Medicine, Technion–Israel Institute of Technology, Haifa, Israel

**Keywords:** COVID-19, health care technology, information management, strategic patient management

## Abstract

The coronavirus disease 2019 (COVID-19) pandemic has remarkably challenged health care organizations and societies. A key strategy for confronting the disease implications on individuals and communities was based on harnessing multidisciplinary efforts to develop technologies for mitigating the disease spread and its deleterious clinical implications. One of the main challenging characteristics of COVID-19 is the provision of medical care to patients with a highly infective disease mandating the use of isolation measures. Such care is complicated by the need for complex critical care, dynamic treatment guidelines, and a vague knowledge regarding the disease’s pathophysiology. A second key component of this challenge was the overwhelming surge in patient burden and the relative lack of trained staff and medical equipment which required rapid re-organization of large systems and augmenting health care efficiencies to unprecedented levels. In contrast to the risk management strategies employed to mitigate other serious threats and the billions of dollars that are invested in reducing these risks annually by governments around the world, no such preparation has been shown to be of effect during the current COVID-19 pandemic. Unmet needs were identified within the newly opened COVID-19 departments together with the urgent need for reliable information for effective decision-making at the state level.

This review article describes the early research and development response in Israel under the scope of in-hospital patient care, such as non-contact sensing of patients’ vital signs, and how it could potentially be weaved into a practical big picture at the hospital or national level using a strategic management system. At this stage, some of the described technologies are still in developmental or clinical evidence generation phases with respect to COVID-19 settings. While waiting for future publications describing the results of the ongoing evidence generation efforts, one should be aware of this trend as these emerging tools have the potential to further benefit patients as well as caregivers and health care systems beyond the scope of the current pandemic as well as confronting future surges in the number of cases.

## INTRODUCTION

The coronavirus disease 2019 (COVID-19) pandemic, caused by the severe acute respiratory syndrome coronavirus 2 (SARS-CoV-2), has spread around the globe during the first half of 2020 and become a leading cause for mortality worldwide. An unprecedented multinational and multidisciplinary effort is being conducted to confront and minimize its implications on individuals and communities.

Health care systems have faced a rapidly spreading infection which required extreme isolation measures on one hand—yet providing mass, extensive, and highly professional critical care treatment amidst a new disease with unknown pathophysiology. While our understanding of the disease is advancing, and treatment guidelines are continuously being rewritten, the mortality rates of admitted patients are still high. The clinical manifestation includes a respiratory distress syndrome sometimes requiring long periods of mechanical ventilation,^1^ accompanied by a cytokine storm. Supportive care is challenging due to the long-term need for mechanical ventilation and the requirement for prone position ventilation and the associated clinical presentation: blood coagulation disorders,^1^ such as venous thrombosis and pulmonary thromboembolic events,^2^ renal failure,^2^ and cardiac manifestations, such as myocarditis with myocardial injury,^2^–^4^ right ventricular dysfunction,^1^ and arrhythmias.^3^ The complex clinical presentation, the extremely large number of patients (around 12.6 million infected worldwide and >562,000 deaths as of July 12, 2020), combined with a prolonged recovery period,^1^ overwhelmed health care systems worldwide.

All these mandated flexibility, agility, and adoption of new protocols and technologies across the board, from the field responders (health care personnel and health care system executives) to governmental agencies and policy makers. Due to the high infection rate, multidisciplinary teams had to adapt quickly and make crucial (and sometimes unpopular) decisions in a short time while basing their decision-making on often unreliable, limited information. Effective response had to be decisive and predicative to the chain of events within a narrow time window, facing an exponential disease spread, while the health of citizens and the economy were at stake.

In contrast to the risk management strategies employed to mitigate military threats, or cyber or terrorist attacks, and the billions of dollars which are invested in reducing these risks annually by governments around the world, no such preparation has been shown to be of effect during the COVID-19 pandemic in most countries.

Unmet needs were identified both within the newly opened COVID-19 departments at the hospitals and within the branches of academia. The threat magnitude, combined with the globally forced economy shut-down, quickly and uniquely brought in strong players with extensive research and development capabilities: civil companies together with army units, large organizations such as army/aviation industries, and small startups—all trying to find solutions to the COVID-19 crisis and its implications.

This review article describes the early research and development response in Israel under the scope of in-hospital patient care, and how it could potentially be weaved into a practical strategic big picture that could help confront the next wave or any upcoming health crisis.

Left out of this discussion are the technologies for disease diagnostics, personal protective gear solutions, and telemedicine aimed at caring for patients outside the hospitals. These are major topics affecting hospital congestion and deserve a separate discussion.

## TECHNOLOGIES AND CHALLENGES

### Vital Signs and COVID-19-Focused Sensors

The risk associated with disease spread to and through health care workers raised the need for telemetry of patients’ status across rooms, often without direct line of sight to some or all patients. The traditional non-invasive measurements of vital signs—blood pressure, heart rate, temperature, respiration rate, and oxygen saturation—had to be transferred to a remote control room. The lack of a sufficient amount of telemetry monitors that would allow a wired or wireless transfer of data to the control room is a key challenge, especially when considering the relatively sudden and unexpected deterioration patterns associated with the disease. This challenge is valid for both critical patients and patients with moderate-to-severe disease. The COVID-19 outbreak facilitated efforts to monitor vital signs without the need for physical contact as well as investigating old and new physiological measurements to allow appropriate patient monitoring.

As body temperature distribution and respiratory dynamics are among the primary indicators for COVID-19 infection,^1^,^5^–^7^ monitoring both these indicators without contact of the subject with a sensor from a distance of a few meters is a key challenge.^8^–^10^ Inferring core body temperature at a medical resolution (an error of about 0.3°C) from afar (more than 2 meters) is not a trivial task as it requires an accurate estimation of the core body temperature based on the detection of the absolute tissue temperature. Similarly, inferring remote and contactless respiratory dynamics is of paramount importance and a major challenge based on current technologies.

Thermal imaging is a method in which a camera’s sensor translates infrared signals into images, which represent the heat emitted from the captured objects.^11^ Uncooled bolometric sensors can sense radiation from objects at a temperature of −40°C to 140°C and can map body and tissue surface temperature remotely. Much research has been conducted in recent years in order to apply thermal imaging techniques for medical uses.^11^ The advantages of thermal imaging as a diagnostic tool arise from the fact that it is a low-cost, non-invasive, safe, and fast method that (most importantly in the COVID-19 case) does not require direct contact with the patient.

Besides fever detection,^10^,^12^ thermal imaging videos were used to infer physiological parameters related to respiratory patterns, such as breathing rate and tidal volume, by monitoring temperature changes around the nostrils.^13^–^15^ An additional use of thermal imaging that is currently under investigation is the assessment of peripheral perfusion. Changes in peripheral perfusion could be used for the detection of pathologies associated with damaged blood flow and for early diagnosis of shock states.^11^ Hand temperature distribution^16^ and foot thermal distribution were used to identify peripheral arterial disease.^17^,^18^ Differences between peripheral organs (ears) and core body temperature^19^ and thermal gradient along the arm^20^ were used for shock diagnosis.

One of the promising approaches to detect respiratory dynamics from a distance involves the usage of Doppler radar.^21^ These radars are not affected by factors such as temperature, light, and humidity but are extremely sensitive to motion and vibration in the vicinity of the subject. To overcome these limitations, a system that includes both Doppler radar and thermal imaging was developed.^22^ Signal integration from both systems was conducted by harnessing machine learning methods. The usage of machine learning occurs on two levels, technical and semantic. On the technical level, it is being used to improve the measurement of the physiological parameters. For example, deep convolutional networks are used for inferring the core temperature based on videos alone without any biophysical model inputs. On the semantic level, clinical status is inferred by aggregating the dynamical data (e.g. the entire body temperature distribution and the respiratory dynamics rather than just common parameters such as core temperature and respiratory rate). The system could be used at the hospital/emergency room entrance in order to test visitors or new patients at a distance without the need for personnel contact, serving as a first contact triage point. The application can allow to similarly trace vital signs in indoor environments (e.g. in emergency rooms). This system was co-developed during the COVID-19 crisis by the Israel Aerospace Industry (IAI) and the Technion and was applied at Rabin Medical Center emergency room, where it was used for remote patient monitoring. Another approach for remote vital sign monitoring was recently suggested by Binah.ai (Ramat-Gan, Israel), an approach relying solely on a smartphone camera either by taking a self-picture or by placing a finger over the camera for a few seconds.^23^

Another system utilizes a piezoelectric sensor integrated into a plate placed under the patient’s bed mattress (EarlySense, Ramat-Gan, Israel).^24^ The device senses the mechanical vibrations and movements created by the patient. Those are analyzed in order to provide contact-free, continuous monitoring of bed-ridden patients and may help in early detection of patient deteriorations.

It is prudent to wait for clinical validation of non-contact vital sign sensing of the above-mentioned systems.

Radar-based sensors (utilizing low-power radio frequency wavelength) are embedded in a system which is used for heart failure management by measuring lung fluid content (ReDS Heart Failure monitor™, Sensible Medical, Netanya, Israel).^25^–^27^ The system measures the dielectric properties of tissues. Low-power electromagnetic signals are emitted through the right mid-thorax, and the signals received after passing through the tissue reflect their combined dielectric properties. These, in turn, are mostly affected by the fluid content of the tissue in the path of the signal. The system is currently being investigated in COVID-19 patients as a tool that could potentially assist in following up the respiratory status of the COVID-19 patients and assist in managing those who require mechanical ventilation.

The high COVID-19 contamination risk, in addition to the shortage of protection gear and its encumbrance, led to a policy of limited entries into contaminated wards (usually one to two times during a 12-hour shift), in order to reduce staff–patient interactions unless an urgent need rises. This new condition in patient care required a new approach. When physicians and nurses contact the COVID-19 patients the heavy protection gear limits visibility and hearing. The use of stethoscope or direct visualization of the nasopharynx is discouraged, thus restricting physical exam to observation alone. The use of existing ultrasound systems (point-of-care ultrasound [POCUS], handheld) to assess patient pulmonary status and diagnose lung pathologies has been reported in COVID-19 patients.^28^,^29^ Audio and/or visually enhanced scopes (Tyto Care, Netanya, Israel) help clinicians evaluate patients’ cardiovascular, pulmonary, and upper respiratory tract status^30^ and enable a complete direct bedside physical exam. This represents an example of how a tool invented and marketed for remote patient home monitoring became relevant as a bedside tool.

### Personnel Command and Control Systems

**Figure 1:**
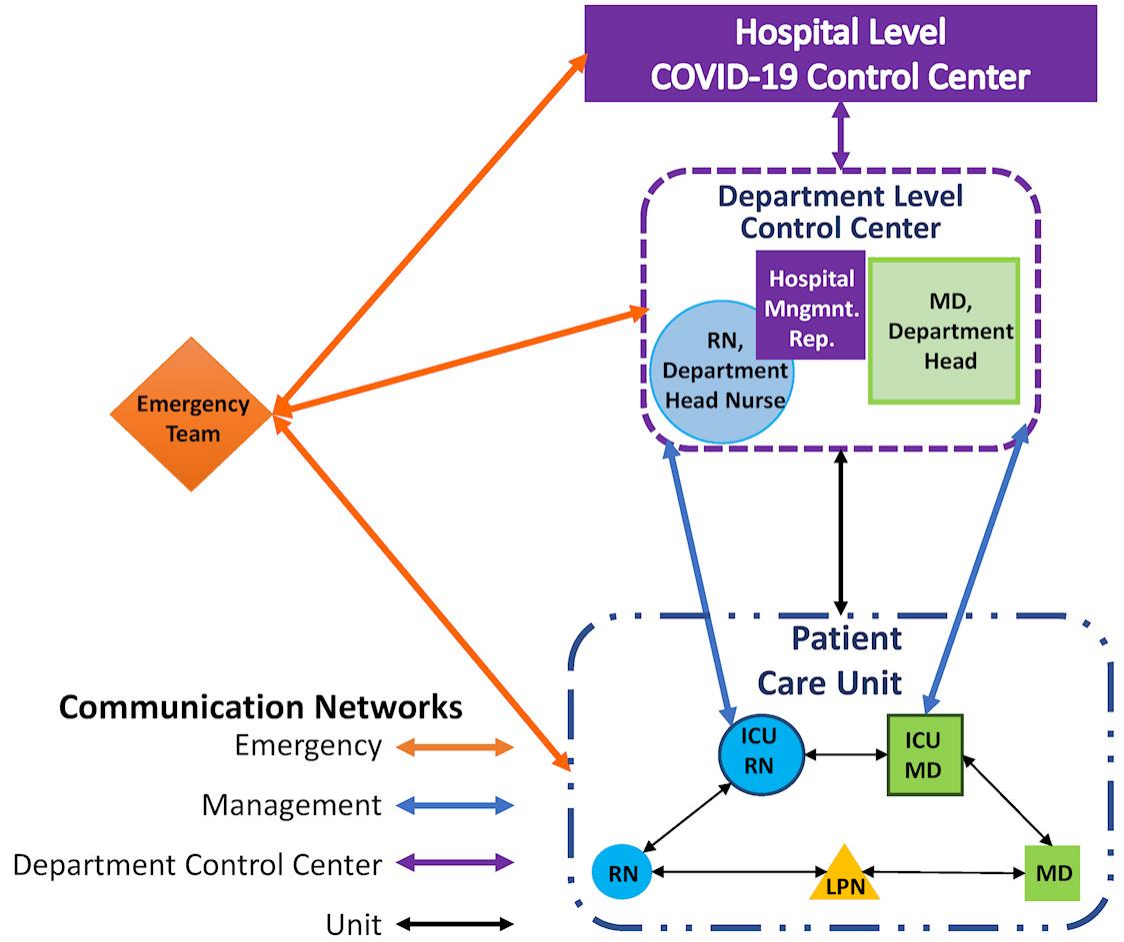
Communication Scheme—EX-TEAMS™ The figure delineates the organized and hierarchal group communication within a COVID-19 department comprising one unit using EX-TEAMS™. The same set-up can be applied to more than one patient care unit within the department. The EX-TEAMS communication network enables communication between the medical staff within the unit (black arrows). The intensive care unit’s MD (ICU MD) and RN (ICU RN) move between the control center and the units, according to clinical needs or get a real time consultation (blue arrows) from within the infected environment (dash-dots blue line) from their colleagues in the control and command center located in a sterile and safe environment (dashed purple line). Each person in the unit, working within the infected environment can also communicate with a hospital management representative (purple box) in the department level control and command center. For every employee in each unit, a location-based system allows calling into action the emergency team and helps them navigate to the exact location where they are needed (orange arrows). In addition, the system allows in-person communication with the paramedical services outside of ExTeams (not shown). ICU, intensive care unit; MD, medical doctor; Mngmnt., management; Rep., representative; RN, registered nurse; LPN, licensed practical nurse.

The expected overwhelming surge of patients during this crisis, the lack of staff trained in the medical intensive care unit (MICU), the requirement for operation within large halls (e.g. underground parking domains) using personal protective equipment (PPE), and the inherent uncertainties associated with a new pandemic may all coalesce to an austere environment with chaotic patterns of communication that may severely hamper the quality of care. Consequently, all entries to a quarantined domain have to be planned and executed flawlessly in order to avoid unnecessary re-entering, much like a spacewalk or entering a battle-zone. Similarly to these scenarios, there is a need for continuous and organized communication between the field teams at the ward or MICU and the control and command center (located in the non-infected environment) that need to integrate and operate the logistics and supportive para-clinical arms. In Israel, combat management strategies, communications, and other technologies were rapidly applied for the COVID-19 environments. Application of such technologies from the battle field to the medical field required mutual adaptations (of both the technology and medical teams) to maximize the operational capabilities of the team. The C^4^I™ system is a command and control tactical system integrating computing, communication, and intelligence information (for health care applications this applies for patient sensors), developed for military use by Elbit Systems. A collaborative effort between Elbit and Rambam Health Care Campus modified the C^4^I system for medical purposes and developed EX-TEAMS ™. EX-TEAMS is a dual WiFi and cellular and cloud-based communication and command platform. It was installed as a software app into push-to-talk mobile phones of all medical and para-clinical teams that operate in the hospital setting caring for COVID-19 patients. A key component of the system is the transformation of medical teams to predefined communication groups organized according to the clinical setting with strict hierarchical communication rules, thereby reducing the levels of the aforementioned chaos (Figure 1). The cornerstone of the system is that each clinical team automatically communicate with the relevant team members using push-to-talk group communication. In addition, the system allows location tracking and automated location-based group switching, thereby allowing each member of the hospital clinical team arriving to a clinical domain to automatically join the relevant clinical team “micro-environment.” The location-based system allows hospital clinical command to better control key personnel (e.g. ICU physicians) and to allocate them according to their location and the clinical need. The addition of a location-based communication may also help to monitor the staff efforts in each clinical section and to allocate resources accordingly and help cut contamination trails if contamination occurs. The system prioritizes response efforts so as to provide efficient mission-focused allocation of workforce and assets, for example rapid assignment and deployment of emergency teams. Additionally, the system allows to receive real-time push-to-video, pictures, peer-to-peer calls, and chats across personnel and between the medical teams and the command and control units. Finally, a key challenge in the setting of COVID-19 is the limited ability to record clinical notes for the electronic medical record (EMR) under PPE. To overcome this challenge, the platform can potentially allow a voice medical record (VMR) to be recorded: a short, “tweet-like,” 1.5-minute voice clip, describing the clinical status of the patient, available to all personnel working in the facility. All the transmitted information is encrypted, maintaining medical confidentiality.

The implementation of the system in COVID-19 departments initially in Rambam and later on in 15 additional hospitals across Israel allowed clinical teams to easily communicate using PPE and to manage, control, and execute the complex tasks performed by a large and diverse workforce operating in a wide range of locations.^31^

Once each ward was equipped with a control room, then (as in usual times) all information available on the patients (directly and indirectly) can be entered into e-records. Connecting all control rooms to a central one at the hospitals and from each hospital to the national level was the next logical step in order to provide a reliable central real-time dashboard.

### Central Command and Control System

During the early phases of the COVID-19 crisis, a real-time hospital command and control tool was evidently needed in order to manage the medical and logistical aspect of the crisis. To maximize hospitals’ efficiency a system integrating patients’ clinical data, medical staff status, critical clinical resources, and asset allocation into a single dashboard was required. The CoView™ System (a collaboration between the Department of Government Medical Centers within the Ministry of Health, the Directorate of Defense Research and Development within the Ministry of Defense, Israel defense forces, and specialists from the hi-tech industry) was developed for this goal, integrating defense concepts, big data analytics, and health care protocols.

**Figure 2:**
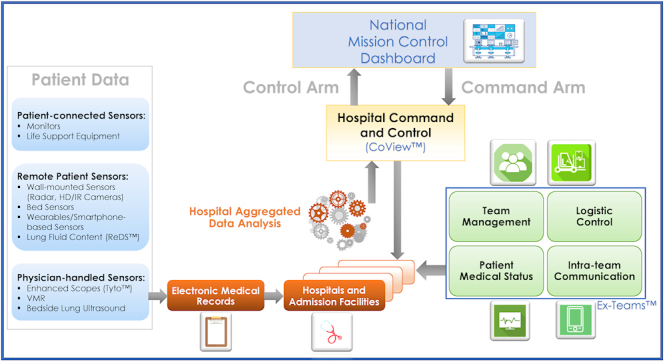
Data Flow Scheme between Teams and Across the Different Operative and Command Levels Different patient data generated by the various sensors/systems (left) and data regarding medical staff competence and critical clinical equipment can be transferred to each local hospital CoView™ system, for each patient record (electronic medical record [EMR]). Patient data from the tactic system (EX-TEAMS™, right) can be uploaded to the relevant patient EMR, while logistics information is uploaded to the relevant administrative databases. The aggregated data (center) can be analyzed at both the hospital and the national levels and presented using a real-time dashboard at each level using the CoView™ platform. The EX-TEAMS system (right) enables tactical organization of the hospital personnel and is used as the operation platform in the organization. The suggested data flow scheme may further facilitate organizational feedback loops to further assist hospitals to perform better and reflect the overall status of the situation to the national decision makers (national mission control dashboard, above). HD/IR, high-definition/infrared; VMR, voice medical record.

The system reflects the status of all COVID-19 patients at all hospitals and admission facilities, in order to enable decision makers to make an efficient, evidence-based, optimized response. Using artificial intelligence algorithms, the system can analyze aggregated data from patient monitors and electronic charts, alerting medical staff to potential deterioration of specific patients, or analyze treatment protocols in specific hospitals (Figure 2). One could centrally control and optimize urgently needed (sometimes scarce or costly) resources such as PPE, drugs, mechanical ventilators, or extracorporeal membrane oxygenation (ECMO) systems. Moreover, professional advisors such as high-level experts can monitor the situation at each hospital, the treatment protocol used, and its efficacy and can help direct hospital staff across the country as needed.^32^,^33^

## CONCLUSIONS AND FUTURE DIRECTIONS

While witnessing the current global surge of cases, health care systems must be prepared also for a potential winter surge with the common flu, a different pandemic, or another health crisis—hopefully with a better readiness than what has been seen so far in most countries during the COVID-19 pandemic. There are many lessons to be learned from the events in our small nation during the past half year. Several conclusions relevant to this discussion should be mentioned:

- Caring for massive numbers of critically ill patients who require strict isolation while maintaining health care personnel safety is challenging and requires “out of the box” thinking.
- A central, real-time overview of hospitals’ status (occupancy, patient condition, logistics, etc.) is fundamental for effective decision-making and resource allocation. This can be helpful to any organization operating during crisis, more so to public health care systems which usually operate on tight budgets and should be prepared for a sudden and extensive stretch in the capacity they need to provide.
- An effective, safe, and widely spread remote patient monitoring capacity is needed for two reasons: to minimize contact between caregivers and patients and to keep hospital beds for the sickest patients who need intensive care. This can be done by keeping the moderately sick patients at home (equipped with sensitive, remote, artificial intelligence-based sensors), while being able to alert whenever and wherever a referral to the hospital is required. In addition, the experience acquired during the COVID-19 pandemic is expected to have a tremendous potential on the continuum of care for chronic disease management like diabetes, heart failure, chronic kidney disease, chronic inflammatory conditions, and high-risk pregnancy.

The number of existing technologies and tools, and those in development, is expected to grow tremendously. As COVID-19’s natural progress proceeds, experience of using such tools and evidence generation needs to be published in order to facilitate a wider adoption of appropriate tools that may benefit patients in the future. For this to succeed, the health care systems and insurers should be prepared with an appropriate infrastructure, including effective reimbursement strategy. The ideal outcome would be for these technologies to provide effective and progressive health care, from the community and regional hospital level to the national level.

## Abbreviations

AI: artificial intelligence
COVID-19: coronavirus disease 2019
ECMO: extracorporeal membrane oxygenation
EMR: electronic medical record
MICU: medical intensive care unit
POCUS: point-of-care ultrasound
PPE: personal protective equipment
SARS-CoV-2: severe acute respiratory syndrome coronavirus 2.
